# Effects of Malnutrition on the Incidence and Worsening of Frailty in Community-Dwelling Older Adults with Pain

**DOI:** 10.3390/nu17091400

**Published:** 2025-04-22

**Authors:** Isabel Rodríguez-Sánchez, José Antonio Carnicero-Carreño, Alejandro Álvarez-Bustos, Francisco José García-García, Leocadio Rodríguez-Mañas, Hélio José Coelho-Júnior

**Affiliations:** 1 Geriatrics Department, Hospital Universitario Clínico San Carlos, 28905 Madrid, Spain; 2 Instituto de Investigación Sanitaria Hospital Clínico San Carlos, 28029 Madrid, Spain; 3 Geriatrics Department, Virgen del Valle Hospital, 45007 Toledo, Spain; lobouc3m@hotmail.com (J.A.C.-C.); franjogarcia@icloud.com (F.J.G.-G.); 4 Biomedical Research Center Network for Frailty and Healthy Ageing (CIBERFES), Institute of Health Carlos III, 28029 Madrid, Spain; a.alvarezbu@gmail.com (A.Á.-B.); leocadio.rodriguez@salud.madrid.org (L.R.-M.); coelhojunior@hotmail.com.br (H.J.C.-J.); 5 Instituto de Investigación IdiPaz, 28029 Madrid, Spain; 6 Geriatrics Department, Getafe University Hospital, 28905 Getafe, Spain; 7 Fondazione Policlinico Universitario Agostino Gemelli IRCCS, 00168 Rome, Italy; 8 Department of Geriatrics, Orthopedics and Rheumatology, Università Cattolica del Sacro Cuore, 00168 Rome, Italy

**Keywords:** musculoskeletal pain, sarcopenia, nutritional status, undernourishment, pain treatment, analgesic, painkillers, opioid

## Abstract

**Background:** Malnutrition may increase the risk of frailty in individuals with musculoskeletal pain. However, this scenario has not been explored in detail. As such, the present study was conducted to examine the effects of malnutrition on the risk of incident and worsening frailty in community-dwelling older adults with musculoskeletal pain. **Methods:** Data from 895 community-dwelling older adults participating in the Toledo Study of Healthy Ageing who reported experiencing musculoskeletal pain during the month preceding data collection (mean age: 74.9 ± 5.6 years) were analyzed. Pain characteristics (i.e., intensity, locations, and treatment) were assessed based on self-reported information regarding the last month. Malnutrition was operationalized according to the GLIM criteria. Frailty status was assessed at baseline and at follow-up (~2.99 years), according to the Frailty Phenotype paradigm, operationalized through the Frailty Trait Scale 5. Associations between the variables were tested using logistic regression analyses adjusted for many covariates established a priori. **Results:** Malnutrition increased the risk of frailty (odds ratio [OR] = 4.41) and worsening of frailty status (OR = 6.25) in the participants who used ≥2 groups of painkillers in comparison to their non-undernourished peers. **Conclusions:** The findings of the present study indicate that malnutrition increases the risk of both developing and worsening frailty in older adults with musculoskeletal disorders. In particular, an increased risk of incident frailty and worsening frailty status was found in undernourished individuals using ≥2 analgesic drugs. Our results suggest that nutritional assessment should be included in the evaluation of old people living with musculoskeletal pain.

## 1. Introduction

Musculoskeletal pain is the leading cause of disability worldwide [1,2]. This condition affects individuals across all age groups, with data estimating that over 600 million people experience musculoskeletal pain around the world [2]. However, its prevalence and severity tend to increase with age, with global estimations indicating that individuals aged 85+ years are more affected [2]. Specifically, among older adults, approximately 30% experience chronic musculoskeletal pain, and 20% suffer from high-impact chronic pain [3]. The development of this condition is multifactorial, often arising from the simultaneous presence of health problems (e.g., osteoarthritis, rheumatoid arthritis, and fibromyalgia) [4] and lifestyle factors (e.g., physical activity levels, smoking) [5,6].

In older adults, the progression of pain is commonly associated with the development of other conditions, such as depressive symptoms, reduced physical performance, and polypharmacy [7,8,9]. These factors may suppress appetite, reducing nutrient intake and causing significant changes in body composition, thereby contributing to malnutrition [10,11]. The presence of malnutrition in older adults is concerning due to its association with adverse outcomes, such as falls, fractures, and mortality [12,13,14], as well as its links to cognitive decline and depressive symptoms [15,16]. As a result, older individuals with both musculoskeletal pain and malnutrition may be at greater risk of poor health outcomes than those with pain alone.

One possible outcome of this problematic combination is frailty. Indeed, numerous observational studies have found that older adults with pain have an increased risk of frailty [17,18,19]. Furthermore, the need for a deeper examination of pain characteristics, including frequency, intensity, and the number of pain sites, has been identified, as specific associations have been observed between these factors and frailty [19].

Malnutrition and frailty share overlapping mechanisms and diagnostic features [6]. Malnutrition affects muscle mass and is linked to the development of functional impairments and sarcopenia [7,8,9], supporting the notion that it is a modifiable risk factor for frailty [6]. However, despite the recognition of the importance of this topic [20], empirical evidence specifically exploring the interplay between malnutrition, pain, and frailty remains scarce.

Based on these premises, the present study analyzed data from the Toledo Study of Healthy Ageing (TSHA) to explore the impact of malnutrition on the risk of frailty in community-dwelling older adults with musculoskeletal pain.

## 2. Materials and Methods

### 2.1. Study Design and Participants

The present study analyzed data from the TSHA database [21]. In summary, the TSHA involved the evaluation of both institutionalized and community-dwelling individuals aged 65+ years living in the province of Toledo, Spain. The TSHA consists of two cohorts. The first, known as the historical cohort, comprises survivors from a previous investigation (the Toledo Study) involving individuals aged 77+ years. The second, the new cohort, includes participants aged 65 to 76 who were newly recruited. Participants from both cohorts were selected using a two-stage random sampling process based on the municipal census of Toledo Province. The sampling was stratified by sex, age, and town size across six strata. The study sample represented approximately 24% of the total population aged 65 years or older in the region.

The study was conducted in accordance with the ethical standards of the Declaration of Helsinki and was approved by the Clinical Research Ethics Committee of Toledo Hospital, Spain (ID: 15072010.93, date of approval of the first protocol: 30 March 2005). All participants provided written informed consent prior to enrollment. The manuscript was prepared in compliance with the Strengthening the Reporting of Observational Studies in Epidemiology (STROBE) guidelines for observational studies [22].

For the present analysis, only participants with complete baseline and follow-up data for the variables of interest were included, with a final sample size of 895 individuals. Baseline and follow-up assessments were conducted between 2011–2013 and 2014–2017, respectively, with a median follow-up of 2.99 years (ranging from 2.0 to 5.4 years).

### 2.2. Study Variables

#### 2.2.1. Musculoskeletal Pain

The presence of musculoskeletal pain in the previous month was assessed based on self-reported information according to three related characteristics. Pain intensity was assessed regarding its impact on daily life activities (DLAs) and classified into the following categories: (a) no pain, (b) mild pain, if it did not interfere with DLAs, and (c) severe pain, when it significantly impacted DLA performance or required sitting or bed rest. The participants could report the presence of pain at four different anatomical sites: (i) hips, (ii) lower back, (iii) knees, or (iv) other joints (e.g., shoulder, elbow, wrist, hands, or ankle). Then, individuals were classified according to the number of pain sites: (a) zero locations, (b) one location, and (c) ≥2 locations. The use of pharmacological treatment for pain was collected according to the Anatomic Therapeutic Chemical (ATC) groups based on the World Health Organization (WHO) classification, as follows: (a) non-steroidal anti-inflammatory drugs (NSAIDs), (b) topical treatment for musculoskeletal pain, (c) analgesics, including opioids and non-opioid pain relievers, (d) antiepileptics, which can also be used for neuropathic pain, and (e) psychoanaleptics, including antidepressants. Then, the analysis was conducted considering the number of ATC groups: (a) no treatment, (b) 1 ATC group, and (c) ≥2 ATC group.

#### 2.2.2. Nutritional Status

Malnutrition was operationalized according to the presence of at least one phenotypic and at least one etiologic criterion, as recommended by the GLIM criteria [23]. Phenotypic criteria included the following parameters: (a) unintentional weight loss: ≥5% in the last 6 months; (b) low body mass index (BMI): <20 kg/m^2^ when <70 years old or <22 kg/m^2^ when ≥70 years old; and (c) reduced isometric grip strength: <30.4 kg for men or 19.8 kg for women, as a supporting measure of reduced muscle mass. Etiologic criteria included (d) reduced food intake, assessed according to the Mini-Nutritional Assessment (MNA)—short form and questions related to the inquiry about protein in the MEDAS questionnaire [24], and (e) inflammation, assessed according to disease burden (i.e., heart failure, dementia, malignant disease, chronic obstructive pulmonary disease, congestive heart failure, chronic renal disease, and diabetes).

#### 2.2.3. Frailty

Frailty status was assessed at baseline and at follow-up according to the Frailty Trait Scale 5 (FTS5) [25]. The FTS5 comprises five domains: gait speed, isometric grip strength, physical activity levels, BMI, and balance. Each domain is scored from 0 (lowest) to 10 (highest), yielding a total score ranging from 0 to 50 points. Individuals scoring > 25 points were classified as frail. Worsening frailty was defined as an increase of ≥2.5 points over time. The FTS5 represents a measure of the phenotypical model of frailty and was selected for being less time-consuming and for outperforming other frailty screening tools (e.g., the Fried Frailty Phenotype) in predicting adverse outcomes across different settings [21,25,26,27,28].

#### 2.2.4. Covariates

The covariates used in the present analysis were established a priori. At baseline, data on age, sex, and educational level were collected. Cognitive function was assessed using the Mini-Mental State Examination (MMSE), with a cut-off score of <23 points suggesting cognitive impairment. Depressive symptoms were evaluated using the 15-item Geriatric Depression Scale (GDS-15), with scores of ≥5 considered indicative of depressive symptomatology. Physical performance was assessed using the Short Physical Performance Battery (SPPB), a hierarchical performance-based evaluation that involves the assessment of balance, walking speed, and lower extremity muscle strength, with final scores ranging from 0 to 12. Physical activity levels were estimated using the Physical Activity Scale for the Elderly (PASE), a validated instrument that captures physical activity levels over a one-week period, encompassing leisure time, household, and occupational activities. PASE scores < 150 are indicative of reduced physical activity levels in older adults.

#### 2.2.5. Statistical Analysis

Data are presented as mean (±standard deviation, SD) for continuous variables and frequency (percentage) for categorical variables. Differences in participants’ characteristics at baseline according to nutritional status were analyzed using Kruskal–Wallis tests for continuous variables and chi-square tests for categorical variables. Logistic regression was conducted to assess the effects of malnutrition on frailty incidence and progression in community-dwelling older adults with musculoskeletal pain. For this purpose, comparisons between undernourished and non-undernourished older adults were conducted to examine changes in frailty status (independent variable) according to pain characteristics (dependent variable). The analysis of the incidence of frailty was adjusted according to age, sex, BMI, cognitive impairment, educational level, depressive symptoms, SPPB, and PASE scores. In contrast, for the analysis of worsening frailty status, BMI, SPPB, and PASE were removed, given that these variables are part of the FTS5. The results of logistic regression are expressed as odds ratios (OR) with corresponding 95% confidence intervals (95% CI). Statistical significance was set at a *p*-value < 0.05. All analyses were conducted using R software, version 4.1.2 (R Foundation for Statistical Computing, Vienna, Austria).

## 3. Results

### 3.1. Main Characteristics of Study Participants

The main characteristics of the participants of the present study according to nutritional status are shown in Table 1. Undernourished participants were older, more prevalently women, with a higher frequency of depressive symptomatology, frailty, lower BMI, physical activity levels, SPPB scores, and higher FTS5 scores. Differences in pain-related characteristics were also observed, with older adults living with malnutrition exhibiting higher rates of severe pain and number of pain sites, except for those with a unique pain location.

### 3.2. Effects of Malnutrition on the Association Between Pain-Related Parameters and the Incidence of Frailty

The incidence of frailty was assessed after a follow-up period of approximately 2.99 years. Undernourished participants exhibited a higher incidence of frailty (17.9%) in comparison to their well-nourished peers (9.5%) (*p* = 0.027). The effects of malnutrition on the association between pain-related parameters and the incidence of frailty are shown in Table 2. In the unadjusted analysis, the presence of malnutrition increased the risk of frailty in the participants who used ≥2 groups of painkillers in comparison to non-undernourished peers. These results remained significant after adjusting for covariates (OR 4.41; 95% CI: 1.05–18.53). No significant associations were observed with either pain intensity or location.

### 3.3. Effects of Malnutrition on the Association Between Pain-Related Parameters and Worsening Frailty

Worsening frailty status was most prevalently observed in malnourished participants (44.3%) compared to non-malnourished individuals (37.6%), although without reaching statistical significance (*p* = 0.31). The effects of malnutrition on the association between pain-related parameters and the worsening of frailty are shown in Table 3. In the unadjusted analysis, undernourished participants using ≥2 groups of painkillers, experiencing severe pain (OR 4.41; 95% CI: 1.06–18.35), and those with ≥2 pain sites (OR 2.08; 95% CI: 1.02–4.24) were at a higher risk of worsening frailty compared to non-undernourished individuals. However, only the associations with those using ≥2 groups of painkillers remained significant after adjusting for covariates.

## 4. Discussion

The present findings indicate that malnutrition modifies the relationship between pain-related parameters and frailty status in community-dwelling older adults. Specifically, undernourished individuals who used two or more classes of analgesic drugs were at increased risk of both developing and worsening frailty compared to their well-nourished counterparts.

Previous studies have consistently shown that malnutrition is a strong determinant of frailty in older adults. Moon et al. [29] identified malnutrition as a major determinant of frailty in older South Korean adults, with undernourished individuals exhibiting a 10-fold increased risk compared to those with normal nutritional status. Similar findings were reported by Wei et al. [30] after examining data of more than 6000 community-dwelling older adults from the Singapore Longitudinal Ageing Study (SLAS). The authors found that older adults with malnutrition were more likely to be frail than those with good nutrition [30]. Bollwein et al. [31] confirmed previous data and provided further alarming evidence by indicating that 90% of undernourished older adults were at risk of frailty. Pooled analyses of the literature confirm the significant associations between malnutrition and frailty [32,33]. In particular, Verlaan et al. [33] examined data of 5447 community-dwelling older adults and reported that frail older adults had an increased risk of being undernourished in comparison to those who were robust.

Our findings expand the current knowledge by highlighting that malnutrition not only affects the frailty burden in robust, well-functioning individuals but also exacerbates the development and progression of frailty in people experiencing musculoskeletal pain [17]. These effects were particularly evident in older adults who were using ≥2 painkillers.

Malnutrition can impact the metabolism of analgesic drugs [34], thereby affecting their pharmacokinetics. For instance, nutrient deficiency may increase the half-life and mean residence time, while reducing the distribution and clearance of drugs like acetaminophen (paracetamol), NSAIDs, and opioids [35,36,37,38].

The pharmacokinetics of analgesic drugs may also be affected by malnutrition-induced changes in body composition. Low lean body mass is associated with reduced total body water and surface area, which in turn decreases the volume of distribution for hydrophilic drugs (e.g., acetaminophen), leading to higher plasma concentrations and an increased risk of toxicity [39,40,41].

An additional pathway linking malnutrition and altered drug metabolism involves the gut microbiota. Several features of malnutrition—such as anorexia and reduced muscle mass—can disrupt microbial balance, promoting dysbiosis. In this context, there is an increase in harmful microorganisms (e.g., pathogenic bacteria) and a decrease in beneficial ones (e.g., probiotics), potentially impairing gut function and its role in maintaining homeostasis [42,43,44,45]. The gut microbiota plays a critical role in drug pharmacokinetics by influencing the metabolism, bioavailability, efficacy, and toxicity of therapeutic agents [46,47]. Moreover, analgesics—particularly opioids and NSAIDs—can exacerbate gut dysbiosis by reducing motility, damaging the intestinal lining, and promoting the overgrowth of pathogenic bacteria [47]. This bidirectional relationship may create a negative feedback loop in which malnutrition-induced dysbiosis alters drug metabolism, further disrupts the gut microbiota, impairs nutrient absorption, and amplifies dysbiosis.

There are several plausible mechanisms that may explain how the association between malnutrition and pain contributes to frailty in older adults. One such mechanism involves the impact of malnutrition on drug efficacy. If analgesics become less effective in undernourished individuals, they may increase their dosage or seek additional pharmacological treatments to manage pain—contributing to polypharmacy. This condition, recognized as a geriatric syndrome, encompasses drug–drug and drug–disease interactions, adverse reactions, anticholinergic burden, and potentially inappropriate prescriptions, all of which are known to promote frailty development [48,49]. The possibility that this scenario explains, at least partially, the results of the present study lies in the fact that malnutrition affects those who are using two or more medications, but not those who are taking only one.

Moreover, changes in the pharmacokinetics associated with malnutrition might contribute to subclinical liver toxicity [35,36]. Over time, such toxicity could impair key physiological systems, such as the cardiovascular, endocrine, and excretory systems, contributing to multimorbidity. This scenario warrants concern, as a nonlinear disruption of multiple physiological systems appears to play a major role in reducing the body’s ability to restore homeostasis after stressful events [50,51].

Additionally, malnutrition and the use of multiple analgesic drugs may exacerbate harmful lifestyle behaviors. Both conditions are associated with inflammation, oxidative stress [20], low protein intake [52], and physical inactivity [53,54], all of which contribute to frailty. Malnutrition is also a known risk factor for sarcopenia [55], which is considered the biological substrate of frailty [56].

These findings have important clinical implications. Specifically, they highlight the need for routine nutritional assessment in older adults with musculoskeletal pain, particularly those using two or more analgesic medications. In individuals who are undernourished but not yet frail, targeted strategies to improve nutritional status may help prevent the onset of frailty. In contrast, for undernourished individuals who are already frail, a more comprehensive approach—addressing nutrition, physical activity, and medication management—may be necessary to mitigate further frailty progression.

The present study has many limitations that should be acknowledged to provide a better interpretation of our results. First, the present analysis is based on community-dwelling older adults, so extrapolations of our findings to individuals in different conditions should be done with caution. Second, the presence of pain was assessed in the last four weeks, which means that complementary analysis taking into consideration other time frames needs to be conducted. Furthermore, no detailed analysis was conducted to examine the etiology of pain, hampering a better description and more specific interpretation of our results. Third, pain characteristics were not assessed using standardized methods, such as the commonly employed numeric rating scale (NRS), visual analogue scale (VAS), verbal rating scale (VRS), or face pain scale. For instance, this scenario limits comparisons to only the two pain intensities examined in the present study, restricting broader conclusions about other levels of pain. Fourth, the inclusion of additional pain scales (e.g., McGill Pain Questionnaire, Pain Catastrophizing Scale) could have enhanced the understanding and depth of our analysis. Fifth, a more detailed analysis of the extension of depressive symptomatology and cognitive deficits could have contributed to a better understanding of our findings. Sixth, the number of participants who developed frailty within specific pain categories may have been too small to detect statistically significant differences. Therefore, more detailed studies employing an a priori power calculation are needed to confirm our findings. Finally, malnutrition was operationalized using muscle strength rather than muscle mass. Although this approach is a valid substitution according to the GLIM criteria, the possibility that different results could be obtained by including muscle mass cannot be ruled out.

## 5. Conclusions

Findings of the present study indicate that malnutrition impacts the incidence and progression of frailty in community-dwelling older adults living with musculoskeletal pain. In particular, increased risk of incident frailty and worsening frailty status were found in undernourished individuals using ≥2 analgesic drugs. These results underscore the importance of including nutritional assessment in the evaluation of older adults living with musculoskeletal pain.

## Figures and Tables

**Table 1:** Main characteristics of study participants according to nutritional status.

Variable	All (*n* = 895)	Non-Malnourished (*n* = 798)	Malnourished (*n* = 97)	*p*-Value
Age, years	74.9 (5.6)	74.6 (5.4)	77.4 (6.1)	0.000
Sex, men	318 (35.5)	300 (37.6)	18 (18.6)	0.003
Cognitive impairment, %	137 (15.3)	117 (14.7)	20 (20.6)	0.157
Educational level, %				
Illiteracy	262 (29.3)	229 (28.7)	33 (34.0)	0.169
Incomplete primary school	274 (30.6)	243 (30.5)	31 (32.0)	
Primary school or higher	359 (40.1)	326 (40.9)	33 (34.0)	
Depressive symptomatology, %	232 (25.9)	184 (23.1)	48 (49.5)	0.000
BMI, kg/m^2^	29.7 (4.7)	29.8 (4.6)	28.7 (5.5)	0.017
FTS5 baseline				
Scores	16.5 (7.0)	16.1 (6.9)	19.4 (7.0)	0.000
Frail, %	101 (11.3)	82 (10.3)	19 (19.6)	0.010
SPPB, mean	8.6 (2.2)	8.6 (2.2)	8.0 (2.4)	0.026
PASE, mean	80.3 (41.0)	81.3 (41.2)	72.1 (39.3)	0.023
*Pain characteristics*				
Intensity, %				
No pain	339 (37.9)	307 (38.5)	32 (33.0)	0.027
Mild pain	475 (53.1)	429 (53.8)	46 (47.4)	
Severe pain	81 (9.0)	62 (7.8)	19 (19.6)	
*Number of pain sites, %*				
Mean (SD)	1.4 (1.2)	1.3 (1.1)	1.7 (1.3)	0.003
0	230 (25.7)	211 (26.4)	19 (19.6)	
1	329 (36.8)	300 (37.6)	29 (29.9)	
2	176 (19.7)	154 (19.3)	22 (22.7)	
3	107 (12.0)	94 (11.8)	13 (13.4)	
4	53 (5.9)	39 (4.9)	14 (14.4)	
*Pain treatment, ATC groups, %*				
Mean (SD)	0.9 (0.8)	0.9 (0.8)	1.0 (0.9)	0.130
0	310.0 (34.6)	282.0 (35.3)	28.0 (28.9)	
1	410.0 (45.8)	364.0 (45.6)	46.0 (47.4)	
≥2	175.0 (19.6)	152.0 (19.0)	23.0 (23.7)	

**Table 2:** Effect of malnutrition on incident frailty according to pain characteristics.

Variable	Unadjusted OR (95% CI)	*p*-Value	Adjusted OR (95% CI)	*p*-Value
Intensity				
Mild pain	1.62 (0.59, 4.46)	0.3476	1.95 (0.60, 6.27)	0.2650
Severe pain	3.33 (0.69, 16.08)	0.1337	5.92 (0.24, 147.76)	0.2790
Pain sites				
1 pain site	1.69 (0.54, 5.28)	0.3662	2.29 (0.61, 8.54)	0.2172
≥2 pain sites	2.18 (0.75, 6.32)	0.1528	2.34 (0.68, 8.07)	0.1798
Pain treatment (ATC groups)				
1 ATC group	2.29 (0.93, 5.68)	0.0729	2.85 (0.93, 8.71)	0.0667
≥2 ATC group	3.50 (1.05, 11.65)	0.0411	4.41 (1.05, 18.53)	0.0427

ATC: anatomic therapeutic chemical; CI: confidence interval; OR: odds ratio.

**Table 3:** Effect of malnutrition on worsening frailty according to pain characteristics.

Variable	Unadjusted OR (95% CI)	*p*-Value	Adjusted OR (95% CI)	*p*-Value
Intensity				
Mild pain	1.28 (0.66, 2.48)	0.4690	1.00 (0.49, 2.06)	0.9909
Severe pain	4.41 (1.06, 18.35)	0.0413	3.98 (0.78, 20.38)	0.0972
Pain sites				
1 pain site	1.38 (0.64, 3.01)	0.4127	0.95 (0.38, 2.37)	0.9161
≥2 pain sites	2.08 (1.02, 4.24)	0.0442	1.74 (0.81, 3.72)	0.1525
Pain treatment (ATC groups)				
1 ATC group	1.12 (0.57, 2.19)	0.7511	0.90 (0.44, 1.84)	0.7766
≥2 ATC group	4.19 (1.48, 11.90)	0.0071	6.25 (1.85, 21.15)	0.0032

ATC: anatomic therapeutic chemical; CI: confidence interval; OR: odds ratio.

## Data Availability

Raw data files are available upon reasonable request.
